# Distinct Symptom Profiles in Younger and Older Patients With Cancer Receiving Chemotherapy

**DOI:** 10.1002/cam4.71652

**Published:** 2026-02-19

**Authors:** Lisa Morse, Sandra Weiss, Christine S. Ritchie, Melisa L. Wong, Bruce A. Cooper, Marilyn J. Hammer, Yvette P. Conley, Steven M. Paul, Jon D. Levine, Christine Miaskowski

**Affiliations:** ^1^ School of Nursing University of California San Francisco California USA; ^2^ Division of Palliative Care and Geriatric Medicine Massachusetts General Hospital Morgan Institute Boston Massachusetts USA; ^3^ Division of Research Kaiser Permanente Medical Group Oakland California USA; ^4^ School of Medicine University of California San Francisco California USA; ^5^ Dana Farber Cancer Institute Boston Massachusetts USA; ^6^ School of Nursing University of Pittsburgh Pittsburgh Pennsylvania USA

**Keywords:** cancer, chemotherapy, latent class analysis, older adults, patient reported outcomes, risk factors, symptom burden, symptoms

## Abstract

**Background:**

Compared to younger patients, older patients report differences in the occurrence, severity, and distress of common symptoms associated with cancer and its treatment.

**Purpose:**

Identify subgroups of younger and older patients with distinct symptom burden profiles and evaluate for risk factors associated with these profiles.

**Methods:**

Oncology outpatients (*n* = 1329) were dichotomized into younger (< 60 years) and older (≥ 60 years) groups. Data included demographic and clinical questionnaires and measures of global, cancer‐specific, and cumulative life stress, resilience, and coping. Memorial Symptom Assessment Scale evaluated the occurrence of 38 common symptoms. Separate latent class analyses were done within each age group to identify distinct symptom profiles. Differences among latent classes in demographic and clinical characteristics, stress, resilience, and coping were evaluated.

**Results:**

In younger group (*n* = 730), four profiles were identified (i.e., All Low (28.8%), Moderate Physical and Lower Psychological (21.9%), Moderate Physical and Higher Psychological (34.6%), All High (14.7%)). Compared to All Low class, All High class was younger, more likely to be female, had a higher comorbidity burden, and a lower functional status, as well as higher stress and lower resilience scores. In the older group (*n* = 599), three profiles were identified (i.e., Low (34.4%), Moderate (47.9%), High (17.7%)). Compared to Low class, High class was more likely to be female, had a higher comorbidity burden and lower functional status, and received a more toxic chemotherapy regimen, as well as higher stress and lower resilience scores.

**Conclusion:**

Study is the first to use latent class analysis to identify distinct symptom burden profiles in younger versus older oncology patients. In the younger group, differences in the occurrence of psychological symptoms differentiated among the symptom burden profiles. While some of the risk factors were similar, within the older group, patients in the High symptom burden class used a higher number of disengagement coping strategies.

## 1 Introduction

A higher symptom burden in oncology patients has a negative impact on treatment adherence [1], functional status [2, 3], and quality of life [1, 4]. Studies report age‐related differences in the occurrence rates of common symptoms associated with cancer and its treatment [5, 6, 7, 8]. Given the increased incidence of cancer among patients 60 years of age and older [9], as well as the limited research that evaluates differences in symptom burden between younger and older oncology patients, a need exists to identify common and distinct risk factors associated with a higher symptom burden in these age groups. One method to determine “symptom burden” is to count the number of symptoms reported by the patient [10, 11, 12]. For example, in a recent cutpoint analysis [11], oncology patients with a low (i.e., 0–8 concurrent symptoms), moderate (i.e., 9–15 concurrent symptoms), and high (i.e., 16–38 concurrent symptoms) symptom burden reported clinically meaningful differences in quality of life.

Several studies demonstrated differences in symptom burden in younger versus older oncology patients using a variety of metrics [5, 6, 7, 8]. For example, in our recent study that utilized the Memorial Symptom Assessment Scale (MSAS) to evaluate age‐related differences in the occurrence of 38 common symptoms associated with cancer and its treatment [5] younger versus older patients reported an average of 15 and 13 concurrent symptoms, respectively. However, relying on the mean number of symptoms may either overestimate or underestimate symptom burden within age groups, making it difficult to identify both younger and older patients who require more intensive symptom management.

Latent variable modeling is an analytic approach that can be used to identify subgroups of patients with distinct symptom burden profiles [13]. Once these profiles are identified, characteristics associated with a higher symptom burden profile can be explored. This approach can assist clinicians to identify high‐risk patients and prescribe age‐appropriate interventions for modifiable risk factors. However, no studies have used latent variable modeling to determine symptom profiles and associated risk factors in younger versus older oncology patients.

### 1.1 Demographic and Clinical Risk Factors Associated With Symptom Burden

Previous studies of oncology patients with heterogenous types of cancer receiving treatment reported that younger age [11, 14, 15, 16], female sex/gender [11, 14, 15, 17, 18], having a higher comorbidity burden [15, 19, 20, 21, 22], a lower functional status [7, 11, 20], a diagnosis of depression [23, 24] and/or a lower income [15, 16] were associated with a higher symptom burden. However, many of these studies had a relatively small proportion of older patients [11, 15, 21, 23] or had exclusion criteria that limited the generalizability of the findings to various types of older patients (e.g., nursing home resident [16], decreased cognitive function [16, 20], not able to carry out most personal needs [14], more than one cancer diagnosis [14, 15], previous or recurrent cancer [17]). Therefore, previous studies may over‐represent younger and more active patients.

### 1.2 Stress and Symptom Burden

Another factor that may contribute to age‐related differences in symptom burden is patients' perceptions of stress [25, 26, 27]. For example, several studies found that younger patients reported higher levels of distress as a result of their cancer diagnosis and associated treatment(s) [28, 29, 30, 31]. In addition, work from our group found that younger age and higher levels of global, cancer‐specific, and/or cumulative life stress were associated with more severe symptoms [25, 26, 32] and a higher symptom burden [33]. For example, in a study that used latent profile analysis [25], three distinct stress profiles were identified (i.e., Stressed (39.3%), Normative (54.3%), Resilient (5.7%)). Oncology patients in the Stressed class were younger; had the highest symptom severity scores; and reported clinically meaningful levels of fatigue, sleep disturbance, and cognitive dysfunction.

However, findings regarding age‐related differences in perceptions of stress are inconsistent. For example, in a study of adult cancer survivors (*n* = 623) that evaluated for an association between perceived stress and the occurrence of chemotherapy‐induced neurotoxicity (i.e., chemotherapy‐induced peripheral neuropathy (CIPN), hearing loss, tinnitus) [34], compared to patients without CIPN, patients with CIPN were significantly older, had higher hyperarousal scores, and higher levels of global stress. While higher levels of stress are associated with a higher symptom burden [33], additional research is warranted on age‐related differences in perceptions of stress and its association with symptom burden.

### 1.3 Resilience and Symptom Burden

A limited amount of research in oncology patients suggests that age influences the relationship between resilience and distress [28, 29]. Psychological resilience refers to a person's capacity to maintain stable emotional functioning when facing stressful life events [35]. Higher levels of resilience support positive adaptation to cancer [36]. In a study of patients with colorectal cancer [28] being older, male, and having fewer cancer‐related problems (i.e., practical, familial, emotional, spiritual, physical) were associated with higher levels of resilience. In addition, higher resilience scores mediated the relationship between older age and lower emotional distress scores. This finding suggests that while older adults experienced lower emotional distress, this effect was largely explained by their higher levels of resilience.

In another study of oncology patients [29], older age was found to moderate the relationship between higher levels of resilience and lower levels of psychological distress. The authors concluded that older patients with higher resilience scores experienced lower levels of psychological distress compared to younger patients with similar levels of resilience. In a study of patients with liver cancer [37], resilience partially mediated the relationship between higher symptom burden and higher distress (i.e., anxiety and depression). This finding suggests that higher resilience slightly reduced the impact of symptom burden on distress. These studies highlight the influential role of resilience in the relationships between age, stress, and symptom burden. However, no studies included measures of resilience in evaluating the relationships between symptom burden and demographic, clinical, and stress characteristics in younger versus older patients with heterogenous types of cancer.

### 1.4 Coping and Symptom Burden

In addition to resilience, the behaviors that patients use to cope with their cancer diagnosis and associated treatments may differ by age [38]. In studies of oncology patients [26, 32, 39, 40, 41], increased use of disengagement coping strategies was associated with a higher symptom burden [39], higher stress scores [26, 32], and higher symptom severity or distress [32, 40, 41]. Disengagement coping strategies are considered to be avoidant‐type behaviors (e.g., denial, venting, substance use, self‐distraction, self‐blame, passive resignation) that enable oncology patients to withdraw from the stressor and/or its associated emotions [42]. In contrast, engagement type coping strategies (e.g., planning, positive reframing, acceptance, humor, religion, emotional support, instrumental support, hope) are action‐oriented behaviors that seek to address the problem or emotion [42].

Only two studies evaluated for differences in coping behaviors between younger and older oncology patients [43, 44]. In one study [44] the most common coping strategy reported by younger patients was hope. In contrast, older patients used passive resignation. In addition, younger patients were more likely to seek social support. In another study [43] compared to older patients with breast cancer, younger patients used more emotional ventilation and experienced greater affective distress. However, these studies used different coping measures, had small sample sizes, and primarily focused on patients with breast cancer. Therefore, additional research is needed to better understand age‐related differences in coping behaviors among patients with heterogeneous types of cancer.

While evidence suggests that age‐related differences exist in the relationships between and among a higher symptom burden and demographic, clinical, stress, resilience, and coping characteristics in patients with cancer, none of these studies performed within‐age group comparisons using a person‐centered analytical approach to characterize symptom burden. Therefore, the purpose of this study was to use separate latent class analyses (LCAs) to identify subgroups of younger and older oncology patients with distinct symptom burden profiles. We hypothesized that within each age group, distinct demographic, clinical, stress, resilience, and coping characteristics would be associated with these symptom burden profiles.

## 2 Methods

### 2.1 Patients and Settings

This longitudinal study is described in detail elsewhere [45, 46]. In brief, eligible patients were ≥ 18 years of age; had a diagnosis of breast, gastrointestinal, gynecological, or lung cancer; had received chemotherapy within the preceding 4 weeks; were scheduled to receive at least two additional cycles of chemotherapy; were able to read, write, and understand English; and gave written informed consent. Patients were recruited from two Comprehensive Cancer Centers, one Veteran's Affairs hospital, and four community‐based oncology programs. A total of 2234 patients were approached and 1343 consented to participate (60.1% response rate). The major reason for refusal was being overwhelmed with their cancer treatment. Of the 1343 patients who consented to participate, 1329 completed the MSAS and had evaluable data for this analysis.

### 2.2 Instruments

#### 2.2.1 Demographic and Clinical Characteristics

A demographic questionnaire obtained information on age, gender, ethnicity, marital status, living arrangements, education, employment status, and income. In addition, patients completed the Karnofsky Performance Status (KPS) scale [47], the Alcohol Use Disorders Identification Test (AUDIT) [48], and the Self‐Administered Comorbidity Questionnaire (SCQ) [49]. A MAX‐2 score was calculated for each patient's chemotherapy regimen. This score is a valid and reliable indicator of the toxicity of various chemotherapy regimens [50].

### 2.3 Symptom Measure

A modified version of the MSAS was used to evaluate the occurrence of 38 symptoms commonly associated with cancer and its treatment. In this study, six symptoms were added to the original list of 32 MSAS symptoms (i.e., hot flashes, chest tightness, difficulty breathing, abdominal cramps, increased appetite, weight gain). Patients were asked to indicate whether or not they had experienced each symptom in the past week (i.e., symptom occurrence). The validity and reliability of the MSAS are well established in oncology patients [51, 52].

### 2.4 Stress, Resilience, and Coping Measures

The 14‐item Perceived Stress Scale (PSS) was used as a measure of global perceived stress according to the degree that life circumstances are appraised as stressful over the course of the previous week [53]. Each item was rated on a 0 to 4 Likert scale (i.e., 0 = never, 1 = almost never, 2 = sometimes, 3 = fairly often, 4 = very often). Total PSS scores can range from 0 to 56. In a probability sample drawn from the Unites States population [54], scores of 18.8 and 20.2 were reported by male and female participants, respectively. Its Cronbach's alpha was 0.89 [55].

The 22‐item Impact of Event Scale‐Revised (IES‐R) was used to measure cancer‐related distress [56] Patients rated each item based on how distressing each potential difficulty was for them during the past week “with respect to their cancer and its treatment.” Three subscales evaluated levels of intrusion, avoidance, and hyperarousal perceived by the patient. The total score can range from 0 to 88. Sum scores of ≥ 24 indicate clinically meaningful post‐traumatic symptomatology and scores of ≥ 33 indicate probable post‐traumatic stress disorder (PTSD) [57]. Cronbach's alpha for the IES‐R total score was 0.92.

The 30‐item Life Stressor Checklist‐Revised (LSC‐R) is an index of lifetime trauma exposure [58]. The total LSC−*R* score is obtained by summing the total number of events endorsed (range of 0 to 30). If the patient endorsed an event, the patient was asked to indicate how much that stressor affected their life in the past year, from one (not at all) to five (extremely). These responses were summed to yield a total “affected” sum score. A PTSD sum score was created based on the number of positively endorsed items (out of 21) that reflect the DSM‐IV PTSD Criteria A for having experienced a traumatic event.

The 10‐item Connor‐Davidson Resilience Scale (CDRS) evaluates a patient's personal ability to handle adversity (e.g., “I am able to adapt when changes occur”; “I tend to bounce back after illness, injury, or other hardships”; and “I believe I can achieve my goals, even if there are obstacles”) [59, 60]. Items are scored on a 5‐point Likert scale (“not true at all” to “true nearly all of the time”). Total scores range from 0 to 40, with higher scores indicative of higher self‐perceived resilience. The normative adult mean score in the United States is 31.8 (±5.4) [61], with an estimated minimal clinically important difference of 2.7 [62]. Its Cronbach's alpha was 0.90.

The 28‐item Brief COPE was used to assess patients' use of 14 coping strategies. Patients rated their use of each coping strategy “since beginning chemotherapy.” Use of each coping strategy was evaluated using 2 items. Each item was rated on a 4‐point Likert scale that ranged from 1 (“I haven't been doing this at all”) to 4 (“I have been doing this a lot”). Scores for each coping strategy can range from 2 to 8, with higher scores indicating greater use of each strategy. Engagement coping strategies and their associated Cronbach's alphas include active coping (0.75), planning (0.74), positive reframing (0.79), acceptance (0.68), humor (0.83), religion (0.92), emotional support (0.77), and instrumental support (0.77). Disengagement coping strategies and their associated Cronbach's alphas include self‐distraction (0.46), denial (0.72), venting (0.65), substance use (0.87), behavioral disengagement (0.57), and self‐blame (0.73) [63].

### 2.5 Study Procedures

The study was approved by the Committee on Human Research at the University of California, San Francisco and by the Institutional Review Board at each of the study sites. Written informed consent was obtained from all patients. Patients completed questionnaires in their homes, a total of six times over two cycles of chemotherapy. Data from the enrollment assessment (i.e., prior to the second or third cycle of chemotherapy) were used in these analyses. Medical records were reviewed for disease and treatment information.

### 2.6 Data Analysis

Patients were dichotomized to the younger (< 60 years) and older (≥ 60 years) groups based on the World Health Organization's reference to the older population as being ≥ 60 years [64]. Separate LCAs were completed for the younger and older groups to identify distinct symptom profiles within each age group. The LCAs were performed using Mplus version 8.1. Estimation was carried out with the maximum likelihood and the expectation maximization algorithms [65]. The optimal number of latent classes for each age group was selected based on the Bayesian Information Criterion (BIC), the Vuong, Lo, Mendel, and Rubin (VLMR) likelihood ratio test, and entropy. In addition, the optimal fitting model should “make sense” conceptually, and its classes should differ as might be expected on variables not used in the generation of the model.

To ensure an adequate number of patients reporting each symptom and to facilitate comparisons with our previous study of the total sample [66], the LCA analyses were done with the 25 symptoms that occurred in ≥ 30% of the patients (i.e., difficulty concentrating, pain, lack of energy, cough, feeling nervous, hot flashes, dry mouth, nausea, numbness or tingling in hands or feet, feeling drowsy, difficulty sleeping, feeling bloated, diarrhea, feeling sad, sweats, problems with sexual interest or activity, worrying, lack of appetite, dizziness, feeling irritable, hair loss, constipation, change in the way food tastes, I do not look like myself, changes in skin). The symptoms that were not included in the LCA were: weight gain, increased appetite, shortness of breath, itching, weight loss, abdominal cramps, mouth sores, difficulty breathing, chest tightness, difficulty swallowing, vomiting, swelling of arms or legs, and problems with urination.

Descriptive statistics and frequency distributions were calculated for demographics and clinical characteristics using Stata, version 18 (StataCorp, College Station, TX). Within each age group, differences among the latent classes in demographic and clinical characteristics, as well as stress, resilience, and coping strategies scores were evaluated using parametric (i.e., analyses of variance) and non‐parametric tests (i.e., Kruskal–Wallis) based on the distribution of the data. Post hoc contrasts were calculated using the Bonferroni procedure (i.e., *p* < 0.0008 for the four class solution (0.05/6) and *p* < 0.017 for the three class solution (0.05/3)).

## 3 Results

### 3.1 Latent Class Solutions

#### 3.1.1 Younger Group

**FIGURE 1:**
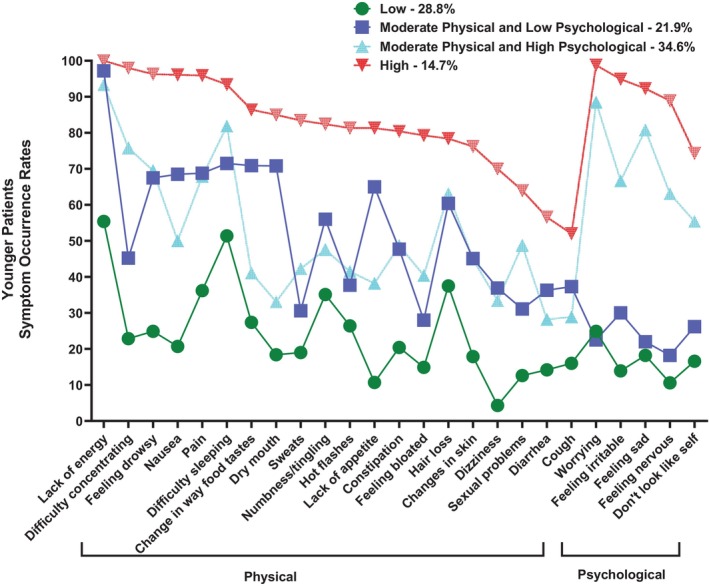
Trajectories of symptom occurrence rates for the four symptom burden classes for the younger oncology outpatients.

In the younger group (*n* = 730), four classes with distinct symptom burden profiles were named: All Low (28.8%), Moderate Physical and Lower Psychological (21.9%), Moderate Physical and Higher Psychological (34.6%), and All High (14.7%) (Figure 1). A four‐class solution was selected because the BIC for that solution was lower than the BIC for the three‐class solution. In addition, the VLMR likelihood ratio test was significant for the four‐class solution, indicating that four classes fit the data better than three classes (Table 1).

**TABLE 1:** Latent class solutions and fit indices using occurrence rates for symptoms on the memorial symptom assessment scale.

Model	LL	AIC	BIC	Entropy	VLMR
Younger patients (< 60 years of age)					
1 Class	−12018.68	24087.36	24202.19	n/a	n/a
2 Class	−11106.73	22315.45	22549.70	0.83	1823.91*
3 Class	−10869.38	21892.75	22246.42	0.84	474.70*
4 Class^a^	−10734.70	21675.40	22148.49	0.80	269.35*
5 Class	−10675.27	21608.54	22201.04	0.80	Ns
Older patients (≥ 60 years of age)					
1 Class	−9497.73	19045.47	19155.35	n/a	n/a
2 Class	−8707.67	17517.35	17741.50	0.85	1580.12*
3 Class^b^	−8556.27	17266.54	17604.98	0.81	302.80*
4 Class	−8477.47	17160.93	17613.64	0.78	ns

*Note:* Baseline entropy and VLMR are not applicable for the one‐class solution.

Abbreviations: AIC, Akaike's Information Criterion; BIC, Bayesian Information Criterion; LL, log‐likelihood; n/a, not applicable; ns, not significant; VLMR, Vuong‐Lo–Mendell–Rubin likelihood ratio test for the K versus K‐1 model.

^a^ The 4‐class solution was selected because the BIC for that solution was lower than the BIC for the 3‐class and 5‐class solutions. In addition, the VLMR was significant for the 4‐class solution, indicating that four classes fit the data better than three classes. In addition, the VLMR for 5‐classes was not significant, indicating that too many classes had been extracted.

^b^ The 3‐class solution was selected because the BIC for that solution was lower than the BIC for the 2‐class and 4‐class solutions. In addition, the VLMR was significant for the 3‐class solution, indicating that three classes fit the data better than two classes. In addition, the VLMR for 4‐classes was not significant, indicating that too many classes had been extracted.

* *p* ≤ 0.001.

As shown in Figure 1, the classes were named based on the probability of occurrence of the 25 MSAS symptoms that occurred in ≥ 30% of the patients. The All Low and All High classes included patients who reported relatively low and high occurrence rates for most of the 25 MSAS symptoms, respectively. The Moderate Physical and Lower Psychological and Moderate Physical and Higher Psychological classes included patients who reported relatively moderate occurrence rates for the majority of the physical symptoms and relatively lower or higher occurrence rates, respectively, for the five psychological symptoms (i.e., worrying, feeling irritable, feeling sad, feeling nervous, I do not look like myself).

#### 3.1.2 Older Group

**FIGURE 2:**
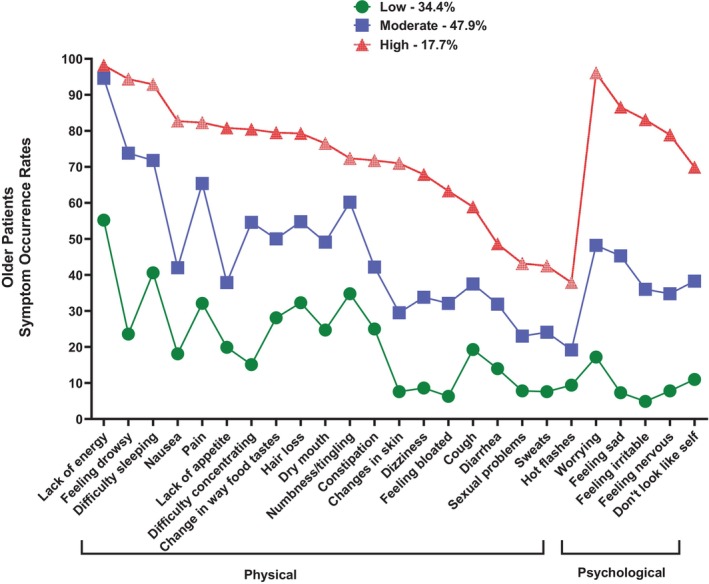
Trajectories of symptom occurrence rates for the three symptom burden classes for the older oncology outpatients.

In the older group (*n* = 599), three classes with distinct symptom burden profiles were named: Low (34.4%), Moderate (47.9%), and High (17.7%) (Figure 2). The BIC for the three‐class solution was lower than the BIC for the two‐class solution. In addition, the VLMR likelihood ratio test was significant for the three‐class solution, indicating that three classes fit the data better than two classes (Table 1). As shown in Figure 2, the Low, Moderate, and High classes included patients who reported relatively low, moderate, and high occurrence rates for most of the MSAS symptoms, respectively.

### 3.2 Demographic and Clinical Characteristics

#### 3.2.1 Younger Group

In the current study, 54.9% of the patients were in the younger group. As shown in Table 2, compared to the All Low and Moderate Physical and Lower Psychological classes, the other two classes were more likely to be female. Compared to the All Low class, the Moderate Physical and Higher Psychological and All High classes were significantly younger. Compared to the All Low and Moderate Physical and Higher Psychological classes, the All High class was less likely to be employed and had a lower annual household income. Compared to the Moderate Physical and Higher Psychological class, the All High class had fewer years of education.

**TABLE 2:** Differences in demographic and clinical characteristics at enrollment among the younger group's symptom burden classes.

Characteristic	All low (0)	Moderate physical and lower psychological (1)	Moderate physical and higher psychological (2)	All high (3)	Statistics
	28.8% (*n* = 210)	21.9% (*n* = 160)	34.6% (*n* = 253)	14.7% (*n* = 107)	
	Mean (SD)	Mean (SD)	Mean (SD)	Mean (SD)	
Age (years)	50.4 (7.9)	48.9 (8.3)	47.0 (9.0)	47.1 (7.8)	*F* = 7.43, *p* < 0.001 0 > 2 and 3
Education (years)	16.2 (3.1)	16.0 (2.9)	16.5 (3.0)	15.4 (2.8)	*F* = 3.56, *p* = 0.014 2 > 3
Body mass index (kg/m^2^)	26.0 (5.5)	26.1 (6.1)	25.9 (5.7)	26.5 (5.7)	*F* = 0.30, *p* = 0.828
Alcohol Use Disorders Identification Test score	3.0 (2.6)	2.4 (1.8)	3.3 (2.8)	2.9 (2.9)	*F* = 2.29, *p* = 0.078
Karnofsky Performance Status score	85.4 (10.7)	78.8 (12.0)	77.1 (11.6)	71.8 (11.2)	*F* = 36.79, *p* < 0.001 0, 1, and 2 > 3 0 > 1 and 2
Number of comorbid conditions	1.7 (1.1)	2.1 (1.2)	2.2 (1.3)	2.8 (1.5)	*F* = 16.44, *p* < 0.001 0, 1 and 2 < 3 0 < 1 and 2
Self‐administered Comorbidity Questionnaire score	3.8 (2.1)	5.1 (2.8)	5.1 (2.9)	6.7 (3.7)	*F* = 25.32, *p* < 0.001 0, 1 and 2 < 3 0 < 1 and 2
Time since diagnosis (years)	1.6 (3.0)	1.2 (2.5)	1.8 (3.5)	1.6 (3.0)	KW = 7.96, *p* = 0.047 1 < 3
Time since diagnosis (median, years)	0.40	0.38	0.40	0.45	
Number of prior cancer treatments	1.5 (1.5)	1.3 (1.4)	1.5 (1.5)	1.8 (1.6)	*F* = 2.76, *p* = 0.041 1 < 3
Number of metastatic sites including lymph node involvement^a^	1.3 (1.3)	1.2 (1.3)	1.2 (1.2)	1.1 (1.2)	*F* = 0.88, *p* = 0.450
Number of metastatic sites excluding lymph node involvement	0.8 (1.1)	0.7 (1.1)	0.7 (1.0)	0.6 (1.0)	*F* = 0.53, *p* = 0.660
MAX2 score	0.18 (0.08)	0.19 (0.08)	0.19 (0.08)	0.18 (0.08)	*F* = 1.31, *p* = 0.269
	% (*n*)	% (*n*)	% (*n*)	% (*n*)	
Gender (% female)	77.1 (162)	78.1 (125)	88.9 (225)	94.4 (101)	*Χ* ^2^ = 24.60, *p* < 0.001 0 and 1 < 2 and 3
Self‐reported ethnicity					*Χ* ^2^ = 18.85, *p* = 0.027
White	62.6 (129)	60.3 (94)	70.5 (177)	54.7 (58)	2 > 3
Asian or Pacific Islander	19.4 (40)	21.2 (33)	10.4 (26)	20.8 (22)	0 and 1 > 2
Black	6.3 (13)	7.7 (12)	5.6 (14)	4.7 (5)	NS
Hispanic, Mixed, or Other	11.7 (24)	10.9 (17)	13.5 (34)	19.8 (21)	NS
Married or partnered (% yes)	69.9 (144)	66.5 (105)	64.3 (160)	59.05 (62)	*Χ* ^2^ = 3.94, *p* = 0.268
Lives alone (% yes)	18.0 (37)	15.2 (24)	18.0 (45)	21.7 (23)	*Χ* ^2^ = 1.83, *p* = 0.608
Currently employed (% yes)	46.9 (97)	40.5 (64)	46.4 (116)	25.2 (27)	*Χ* ^2^ = 16.47, *p* < 0.001 0 and 2 > 3
Annual household income					
Less than $30,000	11.3 (20)	18.6 (27)	13.9 (33)	32.4 (33)	KW = 14.79, *p* = 0.002 0 and 2 > 3
$30,000 to $70,000	18.6 (33)	18.6 (27)	21.1 (50)	15.7 (16)	
$70,000 to $100,000	17.5 (31)	14.5 (21)	15.2 (36)	16.7 (17)	
Greater than $100,000	52.5 (93)	48.3 (70)	49.8 (118)	35.3 (36)	
Childcare responsibilities (% yes)	31.4 (65)	37.5 (57)	35.1 (87)	45.8 (49)	*Χ* ^2^ = 6.58, *p* = 0.087
Elder care responsibilities (% yes)	8.1 (16)	8.6 (12)	8.6 (20)	12.0 (12)	*Χ* ^2^ = 1.37, *p* = 0.713
Past or current history of smoking (% yes)	20.6 (43)	27.1 (42)	29.5 (74)	27.4 (29)	*Χ* ^2^ = 4.97, *p* = 0.174
Exercise on a regular basis (% yes)	77.9 (162)	73.7 (115)	76.8 (189)	65.4 (68)	*Χ* ^2^ = 6.57, *p* = 0.087
Specific comorbid conditions (% yes)					
Heart disease	1.4 (3)	3.8 (6)	1.6 (4)	1.9 (2)	*Χ* ^2^ = 2.99, *p* = 0.393
High blood pressure	18.6 (39)	23.8 (38)	17.8 (45)	22.4 (24)	*Χ* ^2^ = 2.84, *p* = 0.416
Lung disease	5.7 (12)	6.9 (11)	4.7 (12)	10.3 (11)	*Χ* ^2^ = 4.12, *p* = 0.249
Diabetes	5.2 (11)	5.6 (9)	4.7 (12)	6.5 (7)	*Χ* ^2^ = 0.51, *p* = 0.916
Ulcer or stomach disease	3.3 (7)	4.4 (7)	4.3 (11)	9.3 (10)	*Χ* ^2^ = 6.01, *p* = 0.111
Kidney disease	0.5 (1)	1.3 (2)	1.2 (3)	0.9 (1)	*Χ* ^2^ = 0.80, *p* = 0.850
Liver disease	6.2 (13)	6.9 (11)	4.3 (11)	6.5 (7)	*Χ* ^2^ = 1.49, *p* = 0.685
Anemia or blood disease	9.5 (20)	16.3 (26)	16.2 (41)	20.6 (22)	*Χ* ^2^ = 8.05, *p* = 0.045 0 < 3
Depression	5.2 (11)	10.6 (17)	27.3 (69)	42.1 (45)	*Χ* ^2^ = 79.80, *p* < 0.001 0 and 1 < 2 < 3
Osteoarthritis	3.3 (7)	5.6 (9)	7.1 (18)	11.2 (12)	*Χ* ^2^ = 7.92, *p* = 0.048 0 < 3
Back pain	12.9 (27)	25.6 (41)	28.1 (71)	44.9 (48)	*Χ* ^2^ = 39.53, *p* < 0.001 0 < 1 and 2 < 3
Rheumatoid arthritis	1.9 (4)	3.1 (5)	1.6 (4)	3.7 (4)	*Χ* ^2^ = 2.17, *p* = 0.538
Cancer diagnosis					*Χ* ^2^ = 20.74, *p* = 0.014
Breast cancer	47.6 (100)	49.4 (79)	52.2 (132)	53.3 (57)	NS
Gastrointestinal cancer	32.9 (69)	34.4 (55)	21.3 (54)	26.2 (28)	0 and 1 > 2
Gynecological cancer	13.8 (29)	8.1 (13)	20.2 (51)	16.8 (18)	1 < 2
Lung cancer	5.7 (12)	8.1 (13)	6.3 (16)	3.7 (4)	NS
Prior cancer treatment					*Χ* ^2^ = 18.60, *p* = 0.029
No prior treatment	27.9 (57)	35.3 (55)	24.9 (62)	18.9 (20)	1 > 3
Only surgery, CTX, or RT	43.1 (88)	44.9 (70)	46.6 (116)	46.2 (49)	NS
Surgery and CTX, or surgery and RT, or CTX and RT	17.7 (36)	9.6 (15)	16.9 (42)	14.2 (15)	NS
Surgery and CTX and RT	11.3 (23)	10.3 (16)	11.7 (29)	20.8 (22)	NS
Metastatic sites					
No metastasis	34.4 (72)	34.6 (55)	35.6 (89)	41.9 (44)	*Χ* ^2^ = 8.49, *p* = 0.486
Only lymph node metastasis	23.0 (48)	23.9 (38)	23.2 (58)	23.8 (25)	
Only metastatic disease in other sites	13.4 (28)	19.5 (31)	19.2 (48)	13.3 (14)	
Metastatic disease in lymph nodes and other sites	29.2 (61)	22.0 (35)	22.0 (55)	21.0 (22)	
Receipt of targeted therapy					
Only chemotherapy	66.5 (139)	72.7 (112)	73.3 (184)	74.3 (78)	*Χ* ^2^ = 5.11, *p* = 0.530
Only targeted therapy	3.8 (8)	1.3 (2)	3.2 (8)	2.9 (3)	
Both chemotherapy and targeted therapy	29.7 (62)	26.0 (40)	23.5 (59)	22.9 (24)	
Cycle length					
14‐day cycle	45.2 (95)	55.0 (88)	42.5 (107)	47.7 (51)	KW = 6.89, *p* = 0.075
21‐day cycle	48.1 (101)	41.9 (67)	52.4 (132)	47.7 (51)	
28‐day cycle	6.7 (14)	3.1 (5)	5.2 (13)	4.6 (5)	
Emetogenicity of the CTX regimen					
Minimal/low	14.3 (30)	15.0 (24)	19.8 (50)	21.5 (23)	KW = 5.53, *p* = 0.137
Moderate	64.8 (136)	53.1 (85)	56.0 (141)	57.0 (61)	
High	21.0 (44)	31.9 (51)	24.2 (61)	21.5 (23)	
Antiemetic regimen					
None	8.7 (18)	3.8 (6)	6.9 (17)	6.8 (7)	*Χ* ^2^ = 15.55, *p* = 0.077
Steroid alone or serotonin receptor antagonist alone	19.4 (40)	19.6 (31)	20.2 (50)	16.5 (17)	
Serotonin receptor antagonist and steroid	51.5 (106)	50.6 (80)	43.6 (108)	38.8 (40)	
NK‐1 receptor antagonist and two other antiemetics	20.4 (42)	26.0 (41)	29.4 (73)	37.9 (39)	

Abbreviations: CTX, chemotherapy; kg, kilograms; KW, Kruskal–Wallis; m^2^, meters squared; n/a, not applicable; NK‐1, neurokinin‐1; NS, not significant; RT, radiation therapy; SD, standard deviation.

^a^ Total number of metastatic sites evaluated was 9.

Compared to the other three classes, the All High class had a lower KPS score, a higher number of comorbidities, and a higher SCQ score. Compared to the All Low class, the All High class was more likely to self‐report anemia or blood disease and osteoarthritis. Compared to the Moderate Physical and Lower Psychological class, the All High class had a longer time since their cancer diagnosis and a higher number of prior cancer treatments. Among all the classes, self‐reported diagnoses of depression (i.e., All Low and Moderate Physical and Lower Psychological<Moderate Physical and Higher Psychological<All High) and back pain (i.e., All Low<Moderate Physical and Lower Psychological and Moderate Physical and Higher Psychological<All High) had specific patterns.

In terms of cancer type, compared to the All Low and Moderate Physical and Lower Psychological classes, the Moderate Physical and Higher Psychological class was less likely to have gastrointestinal cancer. Compared to the Moderate Physical and Lower Psychological class, the Moderate Physical and Higher Psychological class was more likely to have gynecological cancer (Table 2).

#### 3.2.2 Older Group

The older group consisted of 45.1% of the sample. As shown in Table 3, compared to the Low class, the High class was less likely to be employed and was more likely to self‐report a diagnosis of anemia or blood disease. Compared to the other two classes, the High class had a higher MAX2 score and was more likely to self‐report back pain. Compared to the Low class, the other two classes were more likely to be female, have a diagnosis of breast cancer, receive a highly emetogenic chemotherapy regimen, and were less likely to have gastrointestinal cancer and metastatic disease. Compared to the Low class, the Moderate class was more likely to have care responsibilities for an older adult. Among all the classes, the KPS scores (i.e., Low>Moderate>High), number of comorbid conditions, SCQ scores, and self‐reported depression rates (i.e., Low<Moderate<High) followed specific patterns (Table 3).

**TABLE 3:** Differences in demographic and clinical characteristics at enrollment among the older group's symptom burden classes.

Characteristic	Low (0)	Moderate (1)	High (2)	Statistics
	34.4% (*n* = 206)	47.9% (*n* = 287)	17.7% (*n* = 106)	
	Mean (SD)	Mean (SD)	Mean (SD)	
Age (years)	68.5 (6.0)	68.0 (6.5)	67.3 (5.6)	*F* = 1.25, *p* = 0.287
Education (years)	16.3 (3.1)	16.5 (3.1)	15.7 (3.0)	*F* = 2.10, *p* = 0.123
Body mass index (kg/m^2^)	26.3 (5.4)	26.3 (5.4)	26.9 (6.6)	*F* = 0.50, *p* = 0.607
Alcohol Use Disorders Identification Test score	3.1 (2.1)	2.7 (2.3)	3.4 (3.0)	*F* = 2.18, *p* = 0.115
Karnofsky Performance Status score	86.7 (11.3)	79.7 (12.2)	75.1 (12.3)	*F* = 36.69, *p* < 0.001 0 > 1 > 2
Number of comorbid conditions	2.4 (1.4)	2.8 (1.5)	3.3 (1.7)	*F* = 12.64, *p* < 0.001 0 < 1 < 2
Self‐administered Comorbidity Questionnaire score	5.2 (2.9)	6.3 (3.3)	7.4 (4.0)	*F* = 16.01, *p* < 0.001 0 < 1 < 2
Time since diagnosis (years)	2.2 (3.7)	2.8 (5.2)	2.3 (4.6)	KW = 1.88, *p* = 0.390
Time since diagnosis (median, years)	0.48	0.49	0.42	
Number of prior cancer treatments	1.5 (1.4)	1.8 (1.6)	1.7 (1.2)	*F* = 2.34, *p* = 0.097
Number of metastatic sites including lymph node involvement^a^	1.3 (1.1)	1.4 (1.3)	1.1 (1.2)	*F* = 1.49, *p* = 0.226
Number of metastatic sites excluding lymph node involvement	0.9 (1.0)	0.9 (1.1)	0.7 (1.0)	*F* = 1.33, *p* = 0.265
MAX2 score	0.15 (0.08)	0.16 (0.08)	0.19 (0.07)	*F* = 7.10, *p* < 0.001 0 and 1 < 2
	% (*n*)	% (*n*)	% (*n*)	
Gender (% female)	60.2 (124)	74.1 (212)	79.2 (84)	*Χ* ^2^ = 16.12, *p* < 0.001 0 < 1 and 2
Self‐reported ethnicity				
White	75.9 (154)	79.2 (225)	75.5 (80)	*Χ* ^2^ = 3.31, *p* = 0.769
Asian or Pacific Islander	8.4 (17)	4.9 (14)	8.5 (9)	
Black	7.9 (16)	8.8 (25)	9.4 (10)	
Hispanic, Mixed, or Other	7.9 (16)	7.0 (20)	6.6 (7)	
Married or partnered (% yes)	64.7 (132)	63.1 (178)	58.5 (62)	*Χ* ^2^ = 1.17, *p* = 0.556
Lives alone (% yes)	23.6 (48)	25.9 (73)	31.1 (33)	*Χ* ^2^ = 2.03, *p* = 0.362
Currently employed (% yes)	33.0 (67)	25.2 (72)	18.3 (19)	*Χ* ^2^ = 8.25, *p* = 0.016 0 > 2
Annual household income				
Less than $30,000	17.8 (32)	18.5 (47)	28.4 (27)	KW = 5.31, *p* = 0.070
$30,000 to $70,000	21.1 (38)	25.6 (65)	24.2 (23)	
$70,000 to $100,000	19.4 (35)	17.3 (44)	15.8 (15)	
Greater than $100,000	41.7 (75)	38.6 (98)	31.6 (30)	
Childcare responsibilities (% yes)	6.5 (13)	4.2 (12)	3.0 (3)	*Χ* ^2^ = 2.18, *p* = 0.337
Elder care responsibilities (% yes)	2.6 (5)	9.3 (24)	6.9 (6)	*Χ* ^2^ = 8.04, *p* = 0.018 0 < 1
Past or current history of smoking (% yes)	44.3 (90)	48.8 (137)	45.6 (47)	*Χ* ^2^ = 0.98, *p* = 0.613
Exercise on a regular basis (% yes)	68.3 (140)	67.4 (188)	58.8 (60)	*Χ* ^2^ = 3.06, *p* = 0.217
Specific comorbid conditions (% yes)				
Heart disease	8.3 (17)	11.1 (32)	12.3 (13)	*Χ* ^2^ = 1.59, *p* = 0.451
High blood pressure	43.2 (89)	42.5 (122)	45.3 (48)	*Χ* ^2^ = 0.24, *p* = 0.886
Lung disease	13.6 (28)	19.2 (55)	19.8 (21)	*Χ* ^2^ = 3.13, *p* = 0.209
Diabetes	12.6 (26)	13.9 (40)	15.1 (16)	*Χ* ^2^ = 0.39, *p* = 0.822
Ulcer or stomach disease	2.4 (5)	5.9 (17)	6.6 (7)	*Χ* ^2^ = 4.05, *p* = 0.132
Kidney disease	1.0 (2)	2.1 (6)	3.8 (4)	*Χ* ^2^ = 2.82, *p* = 0.244
Liver disease	6.8 (14)	6.3 (18)	11.3 (12)	*Χ* ^2^ = 3.04, *p* = 0.219
Anemia or blood disease	5.3 (11)	10.1 (29)	14.2 (15)	*Χ* ^2^ = 7.08, *p* = 0.029 0 < 2
Depression	8.3 (17)	21.3 (61)	33.0 (35)	*Χ* ^2^ = 30.10, *p* < 0.001 0 < 1 < 2
Osteoarthritis	18.0 (37)	18.5 (53)	25.5 (27)	*Χ* ^2^ = 2.91, *p* = 0.233
Back pain	18.9 (39)	26.1 (75)	39.6 (42)	*Χ* ^2^ = 15.56, *p* < 0.001 0 and 1 < 2
Rheumatoid arthritis	4.4 (9)	4.9 (14)	2.8 (3)	*Χ* ^2^ = 0.78, *p* = 0.676
Cancer diagnosis				*Χ* ^2^ = 16.74, *p* = 0.010
Breast cancer	18.9 (39)	31.0 (89)	35.8 (38)	0 < 1 and 2
Gastrointestinal cancer	41.7 (86)	30.7 (88)	25.5 (27)	0 > 1 and 2
Gynecological cancer	20.4 (42)	20.9 (60)	18.9 (20)	NS
Lung cancer	18.9 (39)	17.4 (50)	19.8 (21)	NS
Prior cancer treatment				
No prior treatment	25.5 (51)	22.1 (61)	16.8 (17)	*Χ* ^2^ = 4.94, *p* = 0.552
Only surgery, CTX, or RT	38.0 (76)	37.7 (104)	39.6 (40)	
Surgery and CTX, or surgery and RT, or CTX and RT	24.0 (48)	25.0 (69)	31.7 (32)	
Surgery and CTX and RT	12.5 (25)	15.2 (42)	11.9 (12)	
Metastatic sites				*Χ* ^2^ = 17.57, *p* = 0.007
No metastasis	19.9 (40)	31.0 (88)	34.0 (35)	0 < 1 and 2
Only lymph node metastasis	24.9 (50)	16.2 (46)	23.3 (24)	NS
Only metastatic disease in other sites	30.8 (62)	24.3 (69)	25.2 (26)	NS
Metastatic disease in lymph nodes and other sites	24.4 (49)	28.5 (81)	17.5 (18)	NS
CTX regimen				
Only chemotherapy	66.5 (133)	70.2 (198)	67.3 (68)	*Χ* ^2^ = 8.26, *p* = 0.083
Only targeted therapy	5.5 (11)	2.5 (7)	0 (0)	
Both chemotherapy and targeted therapy	28.0 (56)	27.3 (77)	32.7 (33)	
Cycle length				
14‐day cycle	38.3 (79)	34.3 (98)	38.1 (40)	KW = 1.86, *p* = 0.395
21‐day cycle	54.4 (112)	54.2 (155)	50.5 (53)	
28‐day cycle	7.3 (15)	11.5 (33)	11.4 (12)	
Emetogenicity of the CTX regimen				
Minimal/low	20.9 (43)	26.1 (75)	13.3 (14)	KW = 11.66, *p* = 0.003 0 < 1 and 2
Moderate	68.4 (141)	62.0 (178)	64.8 (68)	
High	10.7 (22)	11.8 (34)	21.9 (23)	
Antiemetic regimen				
None	9.9 (20)	7.6 (21)	2.9 (3)	*Χ* ^2^ = 13.16, *p* = 0.041 No significant pairwise contrasts
Steroid alone or serotonin receptor antagonist alone	17.3 (35)	25.4 (70)	21.4 (22)	
Serotonin receptor antagonist and steroid	55.0 (111)	44.2 (122)	49.5 (51)	
NK‐1 receptor antagonist and two other antiemetics	17.8 (36)	22.8 (63)	26.2 (27)	

Abbreviations: CTX, chemotherapy; kg, kilograms; KW, Kruskal–Wallis; m^2^, meters squared; n/a, not applicable; NK‐1, neurokinin‐1; NS, not significant; RT, radiation therapy; SD, standard deviation.

^a^ Total number of metastatic sites evaluated was 9.

### 3.3 Stress and Resilience Characteristics

#### 3.3.1 Younger Group

For the PSS, IES‐R total, and the IES‐R intrusion, avoidance, and hyperarousal subscales, the scores followed the same pattern (i.e., All Low and Moderate Physical and Lower Psychological<Moderate Physical and Higher Psychological and All High; Moderate Physical and Higher Psychological<All High). For the LSC‐R total, LSC‐R affected sum, and the LSC‐R PTSD sum scores, compared to the other three classes, the All High class had higher scores and compared to the All Low class, the Moderate Physical and Higher Psychological class had higher scores. In terms of the CDRS, compared to the All Low and the Moderate Physical and Lower Psychological classes, the other two classes had lower scores (Table 4).

**TABLE 4:** Differences in stress and resilience measures at enrollment among the younger group's symptom burden classes.

Measures^a^	All low (0)	Moderate physical and lower psychological (1)	Moderate physical and higher psychological (2)	All high (3)	Statistics
	28.8% (*n* = 210)	21.9% (*n* = 160)	34.6% (*n* = 253)	14.7% (*n* = 107)	
	Mean (SD)	Mean (SD)	Mean (SD)	Mean (SD)	
PSS total score (range 0 to 56)	15.5 (6.9)	16.7 (7.1)	22.2 (7.6)	25.5 (7.5)	*F* = 63.11, *p* < 0.001 0 and 1 < 2 and 3 2 < 3
IES‐R total sum score					
≥ 24—clinically meaningful PTSD symptomatology	14.2 (10.9)	15.2 (10.9)	22.5 (12.6)	31.2 (16.1)	*F* = 54.09, *p* < 0.001 0 and 1 < 2 and 3 2 < 3
≥ 33—probable PTSD					
IES‐R intrusion	0.6 (0.6)	0.7 (0.6)	1.1 (0.7)	1.5 (0.9)	*F* = 53.92, *p* < 0.001 0 and 1 < 2 and 3 2 < 3
IES‐R avoidance	0.8 (0.7)	0.8 (0.6)	1.0 (0.7)	1.3 (0.8)	*F* = 14.31, *p* < 0.001 0 and 1 < 2 and 3 2 < 3
IES‐R hyperarousal	0.4 (0.5)	0.5 (0.5)	0.9 (0.7)	1.4 (0.8)	*F* = 67.82, *p* < 0.001 0 and 1 < 2 and 3 2 < 3
LSC‐R total score (range 0–30)	5.1 (3.6)	5.7 (3.8)	6.4 (3.8)	7.9 (4.5)	*F* = 10.69, *p* < 0.001 0, 1, and 2 < 3 0 < 2
LSC‐R affected sum score (range 0–150)	9.4 (9.1)	11.6 (12.4)	12.9 (10.4)	17.6 (13.8)	*F* = 10.83, *p* < 0.001 0, 1, and 2 < 3 0 < 2
LSC‐R PTSD sum (range 0–21)	2.4 (2.6)	3.1 (2.9)	3.3 (2.9)	4.5 (3.4)	*F* = 9.68, *p* < 0.001 0, 1, and 2 < 3 0 < 2
CDRS total score					
(31.8 (±5.4)—normative range for the United States population)	31.7 (6.1)	30.7 (6.2)	28.9 (6.3)	27.7 (6.6)	*F* = 12.77, *p* < 0.001 0 and 1 > 2 and 3

Abbreviations: CDRS, Connor Davidson Resilience Scale; IES‐R, Impact of Event Scale – Revised; LSC‐R, Life Stressor Checklist‐Revised; PSS, Perceived Stress Scale; PTSD, post‐traumatic stress disorder; SD, standard deviation.

^a^ Clinically meaningful cutoff scores or range of scores.

#### 3.3.2 Older Group

For the PSS, IES‐R total, and the IES‐R intrusion, avoidance, and hyperarousal subscales, the scores followed the same pattern (Low<Moderate<High). For the LSC‐R total, LSC‐R affected sum, and the LSC‐R PTSD sum scores, compared to the Low class, the other two classes had higher scores. In terms of the CDRS, compared to the other two classes, the High class had lower scores (Table 5).

**TABLE 5:** Differences in stress and resilience measures at enrollment among the older group's symptom burden classes.

Measures^a^	Low (0)	Moderate (1)	High (2)	Statistics
	34.4% (*n* = 206)	47.9% (*n* = 287)	17.7% (*n* = 106)	
	Mean (SD)	Mean (SD)	Mean (SD)	
PSS total score (range 0 to 56)	13.2 (6.6)	18.0 (7.3)	22.2 (8.4)	*F* = 54.98, *p* < 0.001 0 < 1 < 2
IES‐R total sum score				
≥ 24—clinically meaningful PTSD symptomatology	12.0 (9.2)	17.7 (10.6)	27.3 (14.4)	*F* = 64.85, *p* < 0.001 0 < 1 < 2
≥ 33—probable PTSD				
IES‐R intrusion	0.5 (0.5)	0.9 (0.6)	1.3 (0.7)	*F* = 55.52, *p* < 0.001 0 < 1 < 2
IES‐R avoidance	0.7 (0.6)	0.9 (0.6)	1.3 (0.7)	*F* = 23.18, *p* < 0.001 0 < 1 < 2
IES‐R hyperarousal	0.3 (0.4)	0.6 (0.5)	1.1 (0.7)	*F* = 80.19, *p* < 0.001 0 < 1 < 2
LSC‐R total score (range 0–30)	4.9 (2.9)	6.7 (4.0)	6.9 (4.5)	*F* = 64.85, *p* < 0.001 0 < 1 and 2
LSC‐R affected sum score (range 0–150)	8.0 (7.4)	12.6 (10.3)	14.9 (12.2)	*F* = 17.09, *p* < 0.001 0 < 1 and 2
LSC‐R PTSD sum (range 0–21)	2.0 (2.1)	3.5 (3.2)	3.9 (3.7)	*F* = 15.30, *p* < 0.001 0 < 1 and 2
CDRS total score				
(31.8 (±5.4)—normative range for the United States population)	31.2 (6.3)	30.4 (6.0)	28.4 (7.0)	*F* = 6.58, *p* = 0.001 0 and 1 > 2

Abbreviations: CDRS, Connor Davidson Resilience Scale; IES‐R, Impact of Event Scale—Revised; LSC‐R, Life Stressor Checklist‐Revised; PSS, Perceived Stress Scale; PTSD, post‐traumatic stress disorder; SD, standard deviation.

^a^ Clinically meaningful cutoff scores or range of scores.

### 3.4 Coping Strategies

#### 3.4.1 Younger Group

For the use of engagement coping strategies, compared to the All Low class, the Moderate Physical and Higher Psychological class reported higher use of instrumental support. For the other coping strategies (i.e., active coping, planning, positive reframing, acceptance, humor, religion, using emotional support), no statistically significant differences were found among the latent classes (Table 6).

**TABLE 6:** Differences in brief COPE subscale scores at enrollment among the younger group's symptom burden classes.

Subscale^a^	All low (0)	Moderate physical and lower psychological (1)	Moderate physical and higher psychological (2)	All high (3)	Statistics
	28.8% (*n* = 210)	21.9% (*n* = 160)	34.6% (*n* = 253)	14.7% (*n* = 107)	
	Mean (SD)	Mean (SD)	Mean (SD)	Mean (SD)	
Engagement coping strategies					
Active coping	6.2 (1.7)	6.1 (1.6)	6.2 (1.6)	5.8 (1.6)	*F* = 2.16, *p* = 0.092
Planning	5.3 (1.9)	5.3 (1.8)	5.6 (1.7)	5.5 (1.6)	*F* = 1.57, *p* = 0.196
Positive reframing	5.6 (1.9)	5.8 (1.8)	5.7 (1.9)	5.6 (1.9)	*F* = 0.37, *p* = 0.776
Acceptance	6.8 (1.4)	6.8 (1.2)	6.6 (1.3)	6.5 (1.3)	*F* = 1.43, *p* = 0.232
Humor	4.6 (2.1)	4.6 (1.9)	4.7 (1.9)	4.6 (2.2)	*F* = 0.20, *p* = 0.898
Religion	5.0 (2.4)	5.2 (2.3)	5.1 (2.2)	5.6 (2.2)	*F* = 1.78, *p* = 0.150
Using emotional support	6.2 (1.7)	6.4 (1.6)	6.5 (1.6)	6.2 (1.6)	*F* = 1.96, *p* = 0.118
Using instrumental support	5.2 (1.8)	5.4 (1.8)	5.7 (1.7)	5.7 (1.7)	*F* = 4.00, *p* = 0.008 0 < 2
Disengagement coping strategies					
Self‐distraction	5.4 (1.8)	5.4 (1.6)	5.8 (1.6)	5.6 (1.6)	*F* = 3.40, *p* = 0.018 0 < 2
Denial	2.4 (1.1)	2.3 (0.8)	2.7 (1.2)	2.7 (1.3)	*F* = 4.91, *p* = 0.002 1 < 2 and 3
Venting	3.7 (1.5)	3.6 (1.6)	4.7 (1.6)	4.5 (1.6)	*F* = 24.13, *p* < 0.001 0 and 1 < 2 and 3
Substance use	2.2 (0.7)	2.2 (0.7)	2.3 (0.8)	2.3 (0.8)	*F* = 1.70, *p* = 0.167
Behavioral disengagement	2.2 (0.7)	2.1 (0.4)	2.3 (0.7)	2.4 (0.9)	*F* = 4.76, *p* = 0.003 1 < 3
Self‐blame	2.6 (1.0)	2.5 (0.9)	3.3 (1.3)	3.6 (1.8)	*F* = 29.25, *p* < 0.001 0 and 1 < 2 and 3

Abbreviation: SD, standard deviation.

^a^ Each item was rate on a 4‐point Likert scale that ranged from 1 (“I haven't been doing this at all”) to 4 (“I have been doing this a lot”). Each coping strategy is evaluated using 2 items. Scores can range from 2 to 8 with higher scores indicating greater use of each of the coping strategies.

For the use of disengagement coping strategies, compared to the All Low class, the Moderate Physical and Higher Psychological class reported higher use of self‐distraction. For behavioral disengagement, compared to the Moderate Physical and Lower Psychological class, the All High class reported higher scores. For venting and self‐blame, the scores followed the same pattern (i.e., All Low and Moderate Physical and Lower Psychological<Moderate Physical and Higher Psychological and All High) (Table 6).

#### 3.4.2 Older Group

For the use of engagement coping strategies, no statistically significant differences were found among the latent classes. For the use of disengagement coping strategies, compared to the Low class, the other two classes reported higher use of self‐distraction. For behavioral disengagement, compared to the other two classes, the High class reported higher scores. For denial, venting, and self‐blame, the scores followed the same pattern (Low<Moderate<High) (Table 7).

**TABLE 7:** Differences in brief COPE subscale scores at enrollment among the older group's symptom burden classes.

Subscale^a^	Low (0)	Moderate (1)	High (2)	Statistics
	34.4% (*n* = 206)	47.9% (*n* = 287)	17.7% (*n* = 106)	
	Mean (SD)	Mean (SD)	Mean (SD)	
Engagement coping strategies				
Active coping	5.9 (1.8)	5.8 (1.7)	6.0 (1.5)	*F* = 0.28, *p* = 0.757
Planning	4.9 (1.9)	5.2 (1.8)	5.3 (1.8)	*F* = 2.68, *p* = 0.070
Positive reframing	5.0 (2.2)	5.1 (1.9)	5.3 (1.9)	*F* = 0.69, *p* = 0.500
Acceptance	6.8 (1.3)	6.8 (1.3)	6.6 (1.5)	*F* = 1.37, *p* = 0.256
Humor	4.1 (2.0)	4.0 (1.8)	3.6 (1.7)	*F* = 2.16, *p* = 0.117
Religion	5.0 (2.5)	4.6 (2.3)	5.1 (2.3)	*F* = 1.94, *p* = 0.145
Using emotional support	6.3 (1.8)	6.3 (1.7)	6.2 (1.7)	*F* = 0.13, *p* = 0.875
Using instrumental support	4.9 (1.9)	5.1 (1.7)	5.3 (1.6)	*F* = 2.30, *p* = 0.101
Disengagement coping strategies				
Self‐distraction	5.0 (1.9)	5.5 (1.6)	5.6 (1.4)	*F* = 6.51, *p* = 0.002 0 < 1 and 2
Denial	2.2 (0.6)	2.5 (1.1)	2.9 (1.6)	*F* = 15.04, *p* < 0.001 0 < 1 < 2
Venting	3.3 (1.5)	3.8 (1.6)	4.3 (1.7)	*F* = 13.00, *p* < 0.001 0 < 1 < 2
Substance use	2.2 (0.6)	2.2 (0.7)	2.3 (0.9)	*F* = 0.32, *p* = 0.723
Behavioral disengagement	2.2 (0.8)	2.2 (0.8)	2.5 (1.1)	*F* = 4.88, *p* = 0.008 0 and 1 < 2
Self‐blame	2.4 (1.0)	2.7 (1.1)	3.3 (1.4)	*F* = 18.27, *p* < 0.001 0 < 1 < 2

Abbreviation: SD, standard deviation.

^a^ Each item was rated on a 4‐point Likert scale that ranged from 1 (“I haven't been doing this at all”) to 4 (“I have been doing this a lot”). Each coping strategy is evaluated using 2 items. Scores can range from 2 to 8 with higher scores indicating greater use of each of the coping strategies.

## 4 Discussion

This study is the first to use LCA to identify subgroups of younger and older oncology patients with distinct symptom burden profiles. While a four‐class solution was found for the younger group, only three classes were identified for the older group. Age‐related differences in symptom occurrence rates and/or risk factors for a higher symptom burden may explain these differences in the number of distinct symptom burden profiles that were identified.

In the younger group, differences in the occurrence rates for the five psychological symptoms differentiated the symptom burden class assignments. This finding is consistent with our previous analysis of the total sample that identified the same four distinct symptom burden profiles [66]. In addition, the percentage distribution of these symptom burden classes was similar to our findings for the total sample. Compared to the All Low class, the All High class was significantly younger in both analyses. However, in the current study, the mean age of the All High class was slightly younger than in the analysis of the total sample [66] (i.e., 47.1 and 54.4 years, respectively).

While only 17.7% of the older group reported high occurrence rates for the five psychological symptoms, 49.3% of the younger patients had this profile. One possible explanation for this finding is age‐related changes in the activity of the hypothalamic–pituitary–adrenal axis (HPA) [67]. On the one hand, advancing age is associated with the degradation of neuronal cells in the limbic system that leads to dampened adrenocortical diurnal rhythmicity [68] and prolonged elevation of cortisol levels [69, 70]. In addition, neurodegeneration in the hippocampus and prefrontal cortex may impair glucocorticoid receptor‐mediated negative feedback, resulting in a stress response that fails to subside efficiently once activated [71]. These alterations in HPA function may result in allostatic overload, increased emotional reactivity, anxiety, depression, and cognitive impairment [68]. However, chronic stress or prolonged exposure to elevated levels of cortisol can lead to amygdala desensitization or atrophy [72] resulting in blunted emotional responses [73, 74].

While these age‐related changes in the HPA axis provide one explanation for our findings, other interpretations warrant consideration. One potential reason for less distress may be significant decreases in daily life stressors that occur as people age [75] including stressors associated with children, relationships, and work. This hypothesis is supported by a growing body of research that suggests that older individuals experience less psychological distress than younger persons in their lives [76, 77]. In addition, Socioemotional Selectivity Theory suggests that as people age and perceive their remaining time as more limited, their motivational priorities shift toward more meaningful experiences and avoidance of negative stimuli. The net effect is better emotional regulation and prioritization of positive affect [78]. In addition, older patients may experience a response shift due to changes in internal standards and values as they age and expect to experience age‐related declines in their health [79, 80]. For example, the worth they attribute to a symptom like ‘worrying’ may diminish, or they may experience a symptom such as ‘difficulty concentrating’ as a more normal part of life.

Another potential explanation for fewer psychological symptoms in the older patients in the High class may be in the ways that psychological distress presents itself. In a review of depression in older adults [81], a subset of older adults with depression or anxiety reported higher occurrence rates for a number of somatic symptoms (e.g., pain, fatigue, sleep disturbance, dizziness, heart palpitations, gastrointestinal symptoms) compared to affective symptoms (e.g., sadness). In addition, in depressed older adults, a “depression without sadness” phenotype was one that is characterized by non‐dysphoric symptoms (e.g., irritability, apathy, anhedonia, somatic symptoms) [82]. Taken together, changes in the HPA axis, exposure to stressors, life priorities, coping mechanisms, and the appraisal of symptoms may result in lower occurrence rates for psychological symptoms among older patients.

One of the stated aims of this study was to identify common and distinct risk factors for increased symptom burden in younger and older oncology patients. Therefore, the remainder of the discussion compares the current study's findings with the extant literature. Since research on the symptom experience of older oncology patients is limited, the discussion focuses primarily on these adults. Table 8 provides a comparison of the characteristics associated with membership in the higher symptom burden classes for the younger and older age groups.

**TABLE 8:** Characteristics associated with a higher symptom burden in younger versus older oncology patient groups.

Characteristics^a^	Younger patients			Older patients	
	Moderate physical and lower psychological	Moderate physical and higher psychological	All high	Moderate	High
Demographic characteristics					
Younger age		♦	♦		
More likely to be female		♦	♦	♦	♦
Less likely to report being Asian or Pacific Islander		♦			
Less likely to be employed			♦		♦
More likely to have a lower annual household income			♦		
Less likely to report elder care responsibilities				♦	
Clinical Characteristics					
Higher number of comorbidities	♦	♦	♦	♦	♦
Higher Self‐administered Comorbidity Questionnaire score	♦	♦	♦	♦	♦
Lower functional status	♦	♦	♦	♦	♦
Higher MAX2 score					♦
More likely to report a diagnosis of anemia or blood disease			♦		♦
More likely to report a diagnosis of depression		♦	♦	♦	♦
More likely to report a diagnosis of osteoarthritis			♦		
More likely to report a diagnosis of back pain	♦	♦	♦		♦
More likely to have breast cancer				♦	♦
Less likely to have gastrointestinal cancer		♦		♦	♦
More likely to have metastatic disease				♦	♦
More likely to receive a highly emetogenic chemotherapy regimen				♦	♦
Stress characteristics					
Higher Perceived Stress Scale scores		♦	♦	♦	♦
Higher IES‐R intrusion scores		♦	♦	♦	♦
Higher IES‐R avoidance scores		♦	♦	♦	♦
Higher IES‐R hyperarousal scores		♦	♦	♦	♦
Higher IES‐R total scores		♦	♦	♦	♦
Higher LSC‐R total scores		♦	♦	♦	♦
Higher LSC‐R affected sum scores		♦	♦	♦	♦
Higher LSC‐R PTSD sum scores		♦	♦	♦	♦
Lower CDRS scores		♦	♦	♦	♦
Coping characteristics					
Lower instrumental support scores		♦			
Higher self‐distraction scores		♦		♦	♦
Higher denial scores				♦	♦
Higher venting scores		♦	♦	♦	♦
Higher behavioral disengagement scores					♦
Higher self‐blame scores		♦	♦	♦	♦

*Note:* ♦ indicates the presence of the risk factor compared to the Low class for the younger and older patient groups.

Abbreviations: CDRS, Connor Davidson Resilience Scale; IES‐R, Impact of Events Scale‐Revised.

^a^ Comparisons were done with the All Low class for the younger and the Low class for the older patient groups.

### 4.1 Demographic and Clinical Risk Factors

Being female and having a higher comorbidity burden, a lower functional status, and a self‐reported diagnosis of depression were associated with membership in the two highest symptom burden classes for the younger (i.e., Moderate Physical and Higher Psychological and All High) and older (i.e., Moderate and High) age groups. These findings are consistent with previous studies that found being female [11, 14, 17, 18], having a higher comorbidity burden, [19, 20, 21, 83] a lower functional status, [2, 11, 84] and a diagnosis of depression [23, 24] were associated with a higher symptom burden in oncology patients regardless of cancer type, stage of disease, and/or chronological age. In addition, patients in the All High and High classes were less likely to be employed, which may reflect an inability to perform work‐related duties due to their lower functional status and higher comorbidity burden. Of note, compared to the older patients in the High class, younger patients in the All High class had a similar functional status (i.e., KPS scores of 75.1 vs. 71.8, respectively) and comorbidity burden (i.e., SCQ scores of 7.4 vs. 6.7, respectively) despite being, on average, two decades younger. The earlier onset of multimorbidity and functional decline among younger patients in the All High class may be related to an accelerated aging process associated with cancer and its treatment [85]. Additional research is warranted to evaluate this hypothesis.

In terms of depression, approximately 42% of the younger patients in the All High class and 33% of the older patients in the High class self‐reported a diagnosis of depression. For both age groups, these rates are higher than the 8% to 24% range reported in a recent meta‐analysis of the prevalence of depression among oncology patients [86]. The higher prevalence rates for both age groups in the current study may be related to shared biological mechanisms between higher symptom burden and depression (e.g., increased inflammation [87]).

In terms of back pain, in the younger group, compared to the All Low class, a significantly higher percentage of patients in the other three classes reported back pain. However, in the older group, the Low and Moderate classes had similar rates of back pain that were lower than the High class. It is interesting to note that for the All High and High classes, the occurrence rates for back pain were similar (i.e., 44.9% vs. 39.6%, respectively).

For the older patients, a unique risk factor associated with membership in the High class was a higher MAX2 score. This score suggests that these patients received a chemotherapy regimen associated with an increased risk for significant adverse effects [50]. Of note, this class' score was similar to the MAX2 scores for all four classes of the younger patients. This finding may reflect overestimation of treatment tolerance during the clinical decision‐making process. Overtreatment can arise when standard treatment protocols are applied without adequate consideration of patient‐specific vulnerabilities [88]. Best practices recommend that patients ≥ 65 years of age who are receiving chemotherapy have a comprehensive geriatric assessment (CGA) to identify vulnerabilities that are not routinely captured in oncology assessments [89]. Despite best practice recommendations, a recent study found that only 53% of oncology clinicians reported being aware of these guidelines [90].

In addition, conflicting evidence exists on the efficacy of these guidelines in reducing overtreatment of older oncology patients. For example, in a recent study that evaluated predictors of chemotherapy toxicity in older patients with cancer (i.e., sociodemographics, tumor/treatment variables, and geriatric assessment variables), [91] a higher chemotherapy dose (i.e., standard versus reduced) and poorer renal function were the only factors associated with an increased risk for more severe toxicity. Of note, in the current study, the occurrence rates for renal disease were low and creatinine levels at enrollment were within the normal range and did not differ among the symptom burden classes. Taken together, the findings from the current study, along with conflicting evidence regarding the utility of CGA and renal function to identify vulnerabilities to chemotherapy toxicity in older adults, underscore the challenges of delivering effective cancer treatments to these patients while minimizing harm.

### 4.2 Stress and Resilience

In the older age group, a dose response effect was seen for all of the stress measures (i.e., Low<Moderate<High). In contrast, in the younger group, compared to the two lower symptom burden classes, the two higher symptom burden classes had higher but comparable scores for all of the stress measures. However, in both age groups, the All High and High classes had levels of global stress above the normative PSS score for the general population in the United States [54].

In terms of cancer‐specific stress, in the younger group, 40.8% of patients in the Moderate Physical and Higher Psychological class and 28.2% of patients in the All High class had IES‐R total sum scores suggestive of probable PTSD. In the older group, 34.9% of patients in the High class had a IES‐R total sum score suggestive of probable PTSD. These rates are higher than those reported in a meta‐analysis of PTSD in oncology patients (i.e., between 5.1% and 15.3%) [92]. The higher rates reported in our study could be related to the high rates of depression reported among patients in the All High and High groups (i.e., 42.1% and 33.0%, respectively). PTSD and major depressive disorder co‐occur in 52% of patients with PTSD [93].

In addition, 31.7% of the younger patients in the All High and 40.9% of the older patients in the High classes had total IES‐R scores that suggest the occurrence of clinically meaningful PTSD symptomatology. While these findings are consistent with previous reports of overall prevalence rates of between 17% [94] and 44% [95] most studies suggest that younger age is associated with higher rates of PTSD symptomatology [94, 95, 96]. Additional risk factors for PTSD symptomatology include being female [95, 97] having lower educational attainment [97] being unemployed [97] receiving cancer treatment(s) [98] and having a higher number of comorbid conditions [97]. However, these studies did not report findings for younger versus older patients. In the current study, it is notable that most of the risk factors for higher PTSD symptomatology described above were common among the younger and older patients in the worst symptom burden classes. However, additional studies are needed to identify unique risk factors for PTSD symptomatology in older oncology patients because PTSD symptomatology is associated with an increased risk of cognitive impairment [94, 99] and dementia [100]. Clinicians need to assess for PTSD symptomatology in older adults with a high symptom burden using validated screening tools (e.g., Clinician‐Administered PTSD Scale (CAPS) [101] self‐report PTSD Checklist (PCL) [102]) and initiate appropriate interventions to treat PTSD symptoms (e.g., cognitive processing therapy, prolonged exposure therapy) [103].

In terms of lifetime trauma exposure, compared to the All Low and Low classes, the All High and High classes had higher LSC‐R total scores, sum scores for how much stressors affected their lives, and PTSD sum scores. Equally important, these scores were similar between the younger and older patients in the All High and High classes. Given that the All High and High classes differed in age by nearly two decades, these findings raise questions about the timing of the stressful life event(s). Specifically, whether the stressor occurred earlier in life (e.g., an adverse childhood event) or as a result of cancer and its treatment. While types of stressors vary by age [104, 105] understanding the timing and nature of various stressful life events and how these factors differ between younger and older patients are an important foci for future research. Additional studies are warranted because, in oncology patients, experiencing an adverse childhood event is associated with higher levels of inflammation [106], higher symptom burden [106, 107], and higher emotional distress [107]. In addition, understanding age‐related differences in various types of stress may inform the development of targeted interventions to reduce stress for different age groups.

In terms of resilience, in both age groups, compared to the All Low and Low classes, the Moderate Psychological and Higher Psychological and All High (i.e., younger) and High (i.e., older) classes had significantly lower CDRS scores. Of note, for all three classes, the patients' scores were below the normative score for the United States population [61]. It is plausible that the lower occurrence rates of psychological symptoms in the Moderate Physical and Lower Psychological, ALL Low, and Low classes are related to lower levels of stress and higher levels of resilience. Given that previous studies demonstrated that oncology patients with higher levels of resilience experience lower levels of stress [108, 109] and a lower symptom burden [108, 110], both younger and older patients may benefit from interventions that enhance resilience (i.e., positive psychology [111], supportive–expressive group therapy [111], cognitive behavioral therapy [112], mindfulness [112]).

### 4.3 Coping

Adaptive coping styles such as positive reappraisal, social support, religious coping, and spirituality facilitate positive psychological changes that promote the construction of meaning and adaption to stress [113]. In contrast, maladaptive coping can impair problem‐solving and reduce and increase psychological distress [114]. In the current study, membership in the ALL High and High classes was associated with increased use of the disengagement type coping strategies of venting and self‐blame. These findings differ from previous reports [43, 115]. For example, in a study of breast cancer patients [43], as age increased, the use of emotional ventilation as a coping strategy decreased. In another study of patients with a variety of chronic illnesses (e.g., hypertension, diabetes, rheumatoid arthritis, hematologic cancers) [115], compared to middle‐aged adults, older adults were less likely to use emotional expression or self‐blame to cope with their illness. Reasons for these inconsistent findings may be related to the differences in patient characteristics, measures of coping, and timing of the assessments. In addition, variations in levels of stress and resilience between patients in the current study and those in other studies may explain the higher use of venting and self‐blame in the worst symptom burden classes.

Additional disengagement coping strategies used by older adults in the High class included self‐distraction, denial, and behavioral disengagement. These findings are consistent with a study of oncology patients undergoing chemotherapy treatment [44], that reported that compared to younger adults, older adults utilized more passive and/or avoidant coping approaches (e.g., passive resignation). In addition, in a systematic review of qualitative studies that synthesized narratives describing the experiences of older adults who were living with cancer [116], common themes included a sense of disintegration and social retraction. Since disengagement and avoidant type coping behaviors can negatively affect an individual's ability to manage stressors [114], their use among older patients in the High class may reflect poorer adaptation to their cancer diagnosis and may explain the high occurrence of PTSD symptomatology.

However, evidence suggests that coping strategies used by older oncology patients vary across the continuum of cancer care. For example, in a study of older oncology patients [117], during the diagnosis phase, patients relied on a fighting spirit and a determined resolve to adapt to their cancer diagnosis. As these patients transitioned to the treatment phase, a shift toward placing trust in their physicians and seeking social support occurred which suggested a more passive approach to coping with their cancer. In another study of older oncology patients during survivorship [118], coping shifted to more engagement‐type approaches (e.g., social comparison, positive reappraisal, problem‐focused coping, re‐evaluating ordinary events).

While the literature supports oncology patients' use of engagement coping to reduce stress [119] and symptom severity [120] and improve quality of life [121], it is interesting to note that in the younger group, compared to the All Low class, only the Moderate Physical and Higher Psychological class reported an increased use of one engagement coping strategy (i.e., instrumental support). In the older group, no statistically significant differences in the use of engagement coping strategies were found among the latent classes. Additional research is needed to determine whether reducing the use of disengagement coping or increasing the use of engagement coping strategies is more beneficial in decreasing the symptom burden of oncology patients with unique symptom burden profiles and whether these are moderated by age.

Age‐related differences in coping strategies may be related to variations in the type of stress that emerge across the lifespan [122] Equally important, Life Course Theory suggests that people of different ages bring different experiences and resources to situations and may adapt in different ways to new conditions [123]. For example, older age is a period of life that involves confronting mortality and changes in health and function [38]. In addition, declines in executive function [124], lower levels of self‐esteem [125], phase of illness [117, 118], and generational differences in how older oncology patients perceive and participate in their care [44] may influence their use of coping behaviors. Given the paucity of studies, additional research is needed to confirm our findings and determine other factors associated with a higher use of disengagement coping behaviors among older oncology patients with a higher symptom burden. This research is important because disengagement coping is associated with delayed care seeking [126]; lack of adherence with treatment plans [127]; and poorer mental health [128] in older patients without cancer.

### 4.4 Limitations

Several limitations warrant consideration. Because patients were not assessed before the initiation of chemotherapy, whether the risk factors for a higher symptom burden identified in this study were present before the initiation of chemotherapy cannot be determined. Longitudinal studies are warranted that examine age‐related differences in overall symptom burden over time. Since the sample consisted primarily of well‐educated women with limited diversity in self‐reported race and ethnicity, the findings may not generalize to all patients with cancer. Because the most common reason for declining participation was “being overwhelmed by treatment,” the results may underestimate both age groups' levels of stress and symptom burden. However, this large sample of oncology patients undergoing chemotherapy; the evaluation of 25 common symptoms; and the use of LCA within age groups to identify unique symptom burden profiles and associated risk factors are major strengths of this study.

### 4.5 Conclusions and Implications for Research and Practice

Distinct symptom burden profiles in younger versus older oncology patients receiving chemotherapy were identified. While many risk factors for a higher symptom burden were similar across the two age groups, several distinct risk factors for a higher symptom burden in the older group were identified. Both nonmodifiable (e.g., sex, higher comorbidity burden) and potentially modifiable (e.g., back pain, depression, higher perceived and cancer‐specific stress, lower resilience, disengagement coping) risk factors were associated with a higher symptom burden in both younger and older oncology patients. Of note, a risk factor for higher symptom burden among older oncology patients in the High class was the receipt of a more toxic chemotherapy regimen. Clinicians should utilize validated risk assessment tools (e.g., CGA [89]) and emerging models (e.g., Conceptual Model of Treatment Tolerability [129]) to identify older oncology patients with increased risk for chemotherapy toxicity and modify treatment approaches accordingly. The current study provides clinicians with risk factors that can be targeted for interventions.

In addition, a high prevalence of PTSD symptomatology was found among older oncology patients in the worst symptom burden class. Future studies need to identify unique risk factors for PTSD symptomatology in older oncology patients receiving chemotherapy. Clinicians should assess for PTSD symptomatology in older adults with a high symptom burden and initiate appropriate interventions.

Given that both younger and older patients with a high symptom burden had higher levels of global and cancer‐related stress, lower levels of resilience, and higher use of disengagement coping, interventions aimed at enhancing resilience and engagement coping may reduce stress and benefit both groups. Future studies should evaluate whether resilience‐building [111, 112] and engagement‐focused coping [130] interventions can reduce stress and symptom burden in older adults with cancer.

## Author Contributions

**Lisa Morse:** conceptualization (lead); data curation (lead); formal analysis (equal); investigation (equal); methodology (lead); resources (equal); software (equal); validation (equal); writing original draft (lead). **Sandra Weiss:** conceptualization (equal); data curation (equal); investigation (equal); methodology (equal); resources (equal); validation (equal); writing‐review and editing (equal). **Christine S. Ritchie:** conceptualization (equal); data curation (equal); investigation (equal); methodology (equal); resources (equal); validation (equal); writing‐review and editing (equal). **Melisa L. Wong:** conceptualization (equal); data curation (equal); investigation (equal); methodology (equal); resources (equal); validation (equal); writing‐review and editing (equal). **Bruce A. Cooper:** conceptualization (equal); data curation (equal); investigation (equal); methodology (equal); resources (equal); validation (equal); writing‐review and editing (equal). **Marilyn J. Hammer:** conceptualization (equal); data curation (equal); investigation (equal); methodology (equal); resources (equal); validation (equal); writing‐review and editing (equal). **Yvette P. Conley:** conceptualization (equal); data curation (equal); investigation (equal); methodology (equal); resources (equal); validation (equal); writing‐review and editing (equal). **Steven M. Paul:** conceptualization (equal); data curation (equal); investigation (equal); methodology (equal); resources (equal); validation (equal); writing‐review and editing (equal). **Jon D. Levine:** conceptualization (equal); data curation (equal); investigation (equal); methodology (equal); resources (equal); validation (equal); writing‐review and editing (equal). **Christine Miaskowski:** conceptualization (equal); data curation (equal); investigation (equal); methodology (equal); resources (equal); project administration (lead); validation (equal); writing‐review and editing (equal).

## Funding

This work was supported by the National Cancer Institute, CA134900.

## Ethics Statement

This study was approved by the Committee on Human Research at the University of California, San Francisco and by the Institutional Review Board at each of the study sites.

## Conflicts of Interest

The authors declare no conflicts of interest.

## Data Availability

The data that support the findings of this study are available from the corresponding author upon reasonable request.
